# Fusion Validity: Theory-Based Scale Assessment via Causal Structural Equation Modeling

**DOI:** 10.3389/fpsyg.2019.01139

**Published:** 2019-06-04

**Authors:** Leslie A. Hayduk, Carole A. Estabrooks, Matthias Hoben

**Affiliations:** ^1^Department of Sociology, University of Alberta, Edmonton, AB, Canada; ^2^Faculty of Nursing, University of Alberta, Edmonton, AB, Canada

**Keywords:** validity, fusion, scale, structural equation, causal

## Abstract

Fusion validity assessments employ structural equation models to investigate whether an existing scale functions in accordance with theory. Fusion validity parallels criterion validity by depending on correlations with non-scale variables but differs from criterion validity because it requires at least one theorized effect of the scale, and because both the scale and scaled-items are included in the model. Fusion validity, like construct validity, will be most informative if the scale is embedded in as full a substantive context as theory permits. Appropriate scale functioning in a comprehensive theoretical context greatly enhances a scale's validity. Inappropriate scale functioning questions the scale but the scale's theoretical embedding encourages detailed diagnostic investigations potentially challenging specific items, the procedure used to calculate scale values, or aspects of the theory, but also possibly recommends incorporating additional items into the scale. The scaled items should have survived prior content and methodological assessments but the items may or may not reflect a common factor because items having diverse causal backgrounds can sometimes fuse to form a unidimensional entity. Though items reflecting a common cause can be assessed for fusion validity, we illustrate fusion validity in the more challenging context of a scale comprised of diverse items and embedded in a complicated theory. Specifically we consider the Leadership scale from the Alberta Context Tool with care aides working in Canadian long-term care homes.

## Introduction

Scale assessment begins by considering each item's methodology, the respondents' capabilities, and the data gathering procedures (American Educational Research Association, 2014). These fundamental assessments are typically supplemented with evidence of convergent and discriminant validity via factor loadings, factor correlations, and factor score correlations (Brown, 2015). The dependence of factor-based assessments on causal structures is seldom acknowledged, and stands in stark contrast to the causal explicitness accorded typical path models (Duncan, 1975; Heise, 1975; Hayduk, 1987; Bollen, 1989). Combining factor and path structures within programs like LISREL, Mplus, and AMOS encouraged causal understanding of the connections between latent factors and their indicators as well as between different latents (Hayduk and Glaser, 2000a,b; Hayduk et al., 2007; Mulaik, 2010; Hayduk and Littvay, 2012). Including both measurement structure and latent-level structure within a single model makes it possible to investigate what Cronbach and Meehl referred to as construct validity—namely a style of validity assessment grounded in a “nomological network” consisting of an “interlocking system of laws which constitute a theory” where the laws might be “statistical or deterministic” (Cronbach and Meehl, 1955, p. 290). Cronbach and Meehl followed the conventions of their time by replacing cause and causal with synonyms like influences, effects, improves, reflects, results in, and acts on (1955 p. 283–289) but their appeal to “intervening variables” and “specific testable hypotheses” (1955 p. 284, 290) clearly parallel the implications of structural equation models (Hayduk, 1987; Bollen, 1989).

We typically know the full and proximal causal foundations of scale scores because we produce the scale's scores via summing, averaging, weighting, or otherwise combining the values of the items to produce the scale's values. We cause the scale's scores to come into existence by our own, often computer assisted, causal actions. The scale's proximal causal foundations are perfectly known because only the items' recorded values directly determine the scale's values. This causal perfection makes scale scores collinear with the constituent items, and precludes using both the items and scale as data in the same model because the scale scores are seemingly “redundant” with the scale's constitutive items. The fact that the items constitute the full and known proximal causal source of the scale's values does not mean the items' causal sources are known. The values of the items themselves might contain mistakes, inaccuracies, or other features thought of as “error,” but the undetermined causal foundations of the items themselves do not disrupt the causal production of scale scores by summing or averaging the items. We know precisely and perfectly how those scale values came into existence because we the researcher summed, averaged, or weighted the items' values to create the scale scores, and presumably we made no mistakes in these calculations. We know the proximal causes of the scale's values (the items) even though we typically do not know the distal causes of the scale's values (the causes of the items). We also do not know whether the world correspondingly melds or fuses the items' values in the same way we fused the items in forming the scale's values.

This article presents a method for simultaneously modeling both a scale and its constituent items by employing fixed/known effects leading from the items to the scale, and embedding this researcher-dictated causal segment within whatever substantive causally-downstream variables match the researcher's theory about how the scale should function if the world similarly fused or melded the items. The scale is modeled as a latent variable having the items as it's known/fixed causal foundations, without requiring that the scale scores appear in the data. The scale is modeled as an effect of the items, and the items' causes are modeled in accordance with the researcher's understanding of the relevant substantive variables—possibly as the items originating in a common factor (reflective indicators), possibly not (formative indicators) (Bollen and Lennox, 1991).

Including both the items and the scale within a single model permits stronger scale validity assessment because the researcher-dictated causal construction of the scale can be checked for consistency with the world's causal control of the items. Fusion validity extends construct validity by incorporating the known research-production of the scale from the items, into the theory surrounding those items—in full acknowledgment that the world may or may not similarly fuse or meld the items into a corresponding causally-produced and causally-effective scale entity. The dependence of both fusion validity and construct validity on theoretical considerations precludes reducing either fusion validity or construct validity to “a single simple coefficient” (Cronbach and Meehl, 1955, p. 300) but this is multiply recompensed by the substantive considerations addressing whether or not the researcher's constructed scale functions in accordance with the theory-expanded understanding of the world's causal actions.

We detail the relevant procedural steps in the next section, and subsequently illustrate the procedure using the Leadership scale from the Alberta Context Tool (ACT) using data collected in the Translating Research in Elder Care (TREC) program (Estabrooks et al., 2009a,b,c, 2011; https://trecresearch.ca). We address technical and more general issues in concluding sections.

## Methods

### The Logic Underlying Fusion Validity

**Figure 1:**
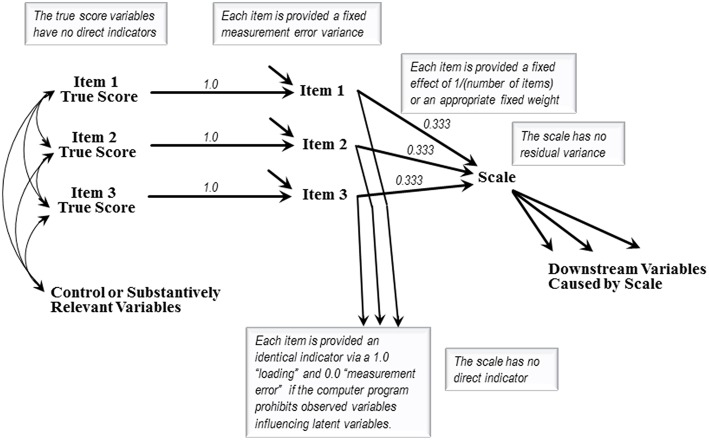
The basic specification of a fusion validity model.

Figure 1 presents the model structure required for assessing the fusion validity of a hypothetical scale calculated as the average of three indicator items. The imagined scale's values are calculated as

$$\begin{array}{lll} Scale & = & \frac{Item1+Item2+Item3}{3}\\ Scale & = & \left(1/3\right)Item1+\left(1/3\right)Item2+\left(1/3\right)Item3\\ Scale & = & 0.333Item1+0.333Item2+0.333Item3. \end{array}$$

The 0.333 coefficients are fixed, not estimated, because the researcher averages the items to causally produce the scale's values. Scales created from weighted items would employ the weights as fixed causal coefficients. Either way the equation producing the scale's values contains no “error” variable because the items in the averaging-equation constitute the complete set of immediate causes of the scale's values.

Figure 1 depicts two causes of each item—an item true score variable, and an unlabeled error variable representing the net impact of all unspecified causes of that item. A fixed 1.0 coefficient causally transmits each case's entire item true score into that case's reported value for the corresponding item. Estimation of the items' true score variances and covariances will be explained below. If freed for estimation an item's measurement error variance will often be underidentified, so these variances will often be fixed based on the literature, or via procedures discussed in Hayduk and Littvay (2012), and retrospectively checked. The items' error sources contribute indirectly to the scale scores even though the scale remains fully causally “accounted for” and has no error variable.

Assessing fusion validity requires embedding a Figure 1 style item-and-scale specification into a model containing one or more substantive variables that are causally downstream from the scale, along with whatever control or substantive exogenous variables the researcher specifies. It is the variables causally downstream from the scale that make estimation possible and that potentially underwrite a scale's fusion validity. The fusion in “fusion validity” concerns whether each item fuses (or mixes/combines/merges/melds) with the other items to form a unidimensional scale-entity absorbing and appropriately dispensing the items' causal consequences. That is, a scale displays fusion validity if the items' causal connections to the downstream variables are adequately modeled by the items having fused into a unidimensional variable displaying theorized effects on the downstream variables. If this causal specification fails to match the data, the validity of the scale is questioned, either because the scale is problematic (the fusing is deficient or incomplete) or because the selected downstream variables were ill advised or improperly modeled.

A model requiring additional effects bypassing the scale by leading directly from an item's true scores to a causally downstream variable is reporting the scale's inability to encapsulate that item's effects. The item's effect transmitted though the scale will require enhancement or reduction if the scale's impact on the downstream variable either over- or under-represents the item's impact. No scale-bypassing effects will be required if the items fuse to form a scale capable of functioning as a full and unitary cause carrying the items' effects to the downstream variables. Researchers can certify the immediate causal foundations of the scale because the researcher is in control the scale's construction, but the world will dictate whether the scaled items' causal capabilities correspondingly combine and fuse. The scale—the putatively fused items—and the individual items' true scores constitute potentially contrasting causal explanations for the items' covariances with the downstream variables.

Fusion validity assessment begins with a ***baseline model*** having only the specified items as causes of the scale, and no effects leading directly from the item true scores to any downstream variables (as depicted in Figure 1). The scale's validity is supported if this specification fits the data and produces anticipated effect estimates. This baseline model implicitly grants the scale preferential treatment because the scale is permitted effects on the downstream variables while any particular item would have to demand a direct effect by disrupting the baseline model's fit until that item is granted its effect. A model that can only be made consistent with the data by permitting an item to have direct scale-bypassing effects is signaling that the scale is unable to fuse or encapsulate the causal impacts of that item. Scale reassessment is required if an ***amended model*** matches the data after supplementation by scale-bypassing effects but whether the scale should be discarded or usefully-retained depends on the revision details. A model remaining inconsistent with the data even after enhancement by scale-bypassing effects, or other alterations, questions whether the downstream and control variables were sufficiently well-understood to underwrite trustworthy scale assessment.

### Examples: Fusion Validity of the Leadership Scale

Our examples employ data from the Translating Research into Elder Care (TREC) archive at the University of Alberta. TREC is a pan-Canadian applied longitudinal (2007-ongoing) health services research program in residential long term care or nursing homes. The TREC umbrella covers multiple ethics-reviewed studies designed to investigate and improve long term nursing-home care (Estabrooks et al., 2009a,c, 2015). We consider the Leadership scale from the Alberta Context Tool which investigates front-line health care aides' perceptions of their care unit work environments. Specifically, we begin with care aide responses to the items comprising the Leadership scale for TREC wave-3 data collected in 2014-2015. The Alberta aides typify the Canadian context by being primarily female (93%), having a first language other than English (61%), and averaging about 46 years of age. We use corresponding Manitoba data to replicate our analysis strategy below, and most Manitoba aides similarly were female (87%), spoke English as a second language (67%), and averaged approximately 45 years of age.

The Leadership scale has undergone traditional measurement assessment (Estabrooks et al., 2009b, 2011) and is calculated by averaging the health care aide's perception of their unit's leader using six 5-point Likert-style items (see Table 1). Specifically the Leadership scale is calculated as the average

$$\begin{array}{l} Leadership\;Scale\\ \;=\frac{Feedback+Success+Calmly+Listens+Mentors+Resolves}{6} \end{array}$$

which corresponds to

$$\begin{array}{l} Leadership\;Scale=\left(\frac{1}{6}\right)Feedback+\left(\frac{1}{6}\right)Success+\left(\frac{1}{6}\right)Calmly\\ \;+\left(\frac{1}{6}\right)Listens+\left(\frac{1}{6}\right)Mentors+\left(\frac{1}{6}\right)Resolves. \end{array}$$

This in turn can be written as an error-free equation containing fixed effect coefficients

$$\begin{array}{l} Leadership\;Scale=\left(0.167\right)Feedback+\left(0.167\right)Success+\left(0.167\right)Calmly\\ \;+\left(0.167\right)Listens+\left(0.167\right)Mentors+\left(0.167\right)Resolves. \end{array}$$

Had the scale been defined as a sum or weighted sum, the fixed values in this scale-producing equation would be either 1.0's or the appropriate item weights.

**Table 1:** Scale items and other variables.

**Items**	**Designation**
**Leadership scale items**	
*The degree to which the aide agrees the identified formal leader of their unit:*	
Looks for feedback even when it is difficult to hear	Feedback
Focuses on successes rather than failures	Success
Calmly handles stressful situations	Calmly
Actively listens, acknowledges, and then responds to	Listens
requests and concerns	
Actively mentors or coaches performance of others	Mentors
Effectively resolves conflicts that arise	Resolves
**Other variables**	
I am a member of a supportive work group	Supportive
I have control over how I do my work	Control
My observations about resident conditions are routinely	Taken
taken seriously by those in positions of authority	
I am comfortable talking about resident care issues with	Talk
those in positions of authority	
How often do you have time to do something extra for	Extra
residents	
In general, I like working here	Like Work Here
I feel burned out from my work	Burnout
Sex	Sex
Age	Age
English first language	English
For-profit organization	Profit
We have enough staff to get necessary work done	Staff
Number out of six possible kinds of resident reactive	Aggressive
behaviors experienced in the last 5 shifts	

*The Leadership scale is the average (mean) of the six Leadership items. The “Other Variables” are single response items, some of which are defined as contributing to scales in other contexts*.

*Most items are scored 1 = strongly disagree, 2 = disagree, 3 = neither agree nor disagree, 4 = agree, 5 = strongly agree*.

*Extra is scored 1 = never, 2 = rarely, 3 = occasionally, 4 = frequently, 5 = almost always*.

*Sex: 1 = male, 2 = female*.

*Age: in decade-delimited years*.

*English: 1 = English first language, 0 = Other first language*.

*Profit: 1 = working in a for-profit organization, 0 = working in a not-for-profit organization*.

**Figure 2:**
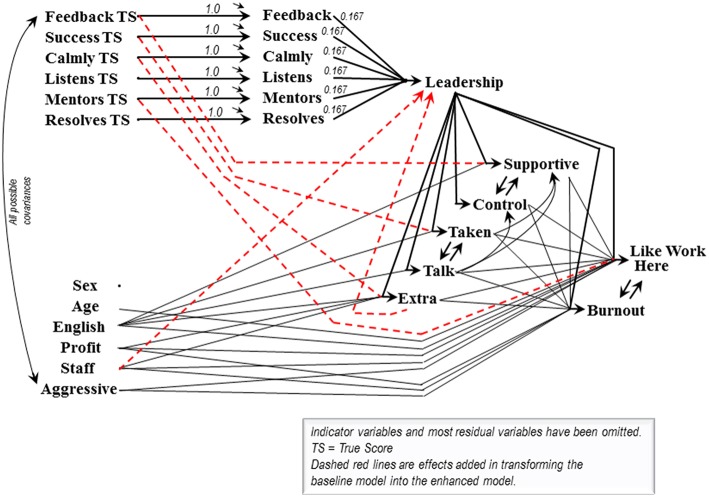
Leadership baseline and enhanced models for Alberta.

Figure 2 depicts the production of the Leadership scale, along with the effects of Leadership on several interrelated downstream variables. The attitudinal indicators of the downstream variables and the items comprising the scale are each assigned 5% measurement error variance in the models we consider. The exogenous control variables are assigned the following measurement error variances: Sex 1%, Age 5%, English as first language 5%, For-Profit organization 0%, Enough Staff 5%, and Aggressive acts (negative resident behavioral responses) 5%. The leadership items' measurement errors are included at the latent level of the model to correspond to routine construction of scales from error-containing items rather than from item true scores.

Assessing a scale's fusion validity begins with a ***baseline***model, and may or may not require construction of an ***amended***model. The baseline model includes:

the items' contributions to the scale,
the scale's effects on the downstream variables,
any effects among the downstream variables,
the control variables' covariances with the scale items
and the control variables' theorized connections to the downstream variables,

but

*no* direct effects of the items on the downstream variables,
and *no* effects leading directly to the scale (beyond the scale's items).

A baseline model displaying clean fit and theory-consistent estimates supports the scale's validity. Item effects bypassing the scale, or additional effects leading to the scale, may appear in an amended model but such effects constitute evidence recommending scale reassessment. Syntax for both the baseline and amended Leadership models is provided near the end of this article.

Both the baseline and amended models might fit or fail to fit, but even a failing baseline model should provide somewhat-reasonable estimates because wild baseline estimates potentially indicate the scale is being encumbered by non-sensical theory-claims about the scale's connections to the downstream variables. Limited modifications to the baseline model are permitted if they maintain the features listed above but such modifications should respect and preserve evidence more appropriately seen as questioning the scale's construction. The modifications to the baseline Leadership model for the Alberta data were minimized and fastidiously critiqued (by *LH*) because we planned to subsequently employ the same baseline model with Manitoba data. The objective here was ***not*** to attain fit, but to ensure that the portions of the model concerning the downstream and control variables provided a reasonable theory-context for the Leadership scale. In fact, the resultant Alberta baseline Leadership model remained highly significantly ill fitting (χ^2^ = 199.0, *df* = *67, p* = 0.000, see Table 2), suggesting the Leadership scale does not adequately fuse or encapsulate the causal impacts of the leadership items. The baseline model retained all the initially postulated effects whether significant or insignificant. Insignificant estimates constitute unfulfilled theory expectations but they also constitute a cataloged theory-reserve potentially buttressing modifications introduced during construction of an amended model.

**Table 2:** Model tests.

	**χ^**2**^**	**df**	***P***
Alberta baseline	199.0	67	0.000
Alberta amended	70.5	61	0.189
Manitoba baseline	113.9	68	0.000
Manitoba amended	82.8	66	0.079

*χ^2^ = chi-square*,

*df = degrees of freedom*,

*P = probability*.

Amending a failing baseline model focuses on additional effects emanating from the items and/or effects leading to the scale—namely the effects expressly excluded from the baseline model. Additional item effects will usually originate in the item true-scores because the measurement errors contributing to the observed items are not expected to impact downstream variables. Coefficients suggested by the modification indices were considered individually and added sequentially, based on the *post-hoc* theoretical palatability of their signs, magnitudes, and modeling consequences (such as avoiding underidentification) but for brevity we proceed as if six effects (detailed in the Appendix model syntax) were added simultaneously to create the enhanced Leadership model. The amended model fits according to χ^2^ with *p* = *0.19* (Table 2) and provides the estimates in Table 3. The baseline and amended models permit seven possible direct Leadership-scale effects on the downstream variables. All seven estimates were in the anticipated direction, and five were significant, but these effects do not accurately portray the full effectiveness of some of the items on the downstream variables. Four of the six coefficients added in forming the amended model are item effects bypassing the Leadership scale by leading directly from an item's true score to a downstream variable. The effects are: Feedback to Supportive Group, Success to Observations Taken Seriously, Calmly to Time for Something Extra, and Leader Mentors to Like Working Here. These effects lead from four different items' true scores to four different downstream variables and hence cannot be dismissed as artifacts created by a single problematic item.

**Table 3:** Amended leadership model.

		**Supportive**	**Control**	**Taken**	**Talk**	**Extra**	**Like Here**	**Burnout**	**Leadership**	**Sex**	**Age**	**English**	**Profit**	**Staff**	**Aggressive**	**TS Feedback**	**TS Success**	**TS Calmly**	**TS Listens**	**TS Mentors**	**TS Resolves**	**R^**2**^**
Supportive	AB		−0.388^*^	0.224^*^	0.148^*^				0.473^*^			0.236^*^				−0.102^*^						0.324
	MB		−0.150	0.099^*^	0.104^*^				0.499^*^			0.105										0.219
Control	AB	0.556^*^			0.090^*^				0.103													0.331
	MB	0.332^*^			0.101^*^				0.257^*^													0.215
Taken	AB				0.167^*^				0.455^*^			0.216^*^					−0.084^*^					0.226
	MB				0.169^*^				0.627^*^			0.075						−0.115^*^				0.241
Talk	AB			0.167^*^					0.191^*^			−0.145^*^										0.080
	MB			0.169^*^					0.129^*^			−0.110										0.059
Extra	AB								0.412^*^			0.321^*^	0.207^*^	0.242^*^				−0.206^*^				0.217
	MB								0.160^*^			0.435^*^	0.309^*^	0.250^*^								0.168
Like Here	AB	0.115^*^	0.120^*^	0.141^*^	0.032	0.083^*^		−0.062^*^	0.123^*^		0.026^*^	0.041	−0.047	0.027	0.001					0.082^*^		0.355
	MB	0.140^*^	0.064^*^	0.114	0.030	0.036		−0.062^*^	0.098		0.026^*^	0.013	−0.182^*^	0.069^*^	−0.005							0.265
Burnout	AB	−0.152^*^	0.049	−0.079	−0.086	−0.165^*^	−0.062^*^		−0.098				0.137	−0.367^*^	−0.194^*^							0.153
	MB	−0.377^*^	−0.086	−0.066	−0.002	−0.164^*^	−0.062^*^		0.181				0.088	−0.324^*^	−0.229^*^							0.137
Leadership	AB					0.099^*^								0.173^*^								1.00
	MB													0.148^*^								1.00

*The fixed 1.0 and 0.167 coefficients leading to and from the items are not shown*.

*Alberta N = 1610, Manitoba N = 744. Alberta Browne's χ^2^ = 70.5, df = 61, p = 0.19. Manitoba Browne's χ^2^ = 82.8, df = 66, p = 0.08*.

*AB, Alberta; MB, Manitoba; TS, True Score*.

*Coefficients are unstandardized maximum likelihood estimates from LISREL 9.1 (Joreskog and Sorbom, 2016)*.

*Coefficients in highlighted italics were added in forming the amended model, and the −0.150 effect of Control on Supportive in the MB model was fixed at a researcher-assessed value to ensure identification*.

* *Indicates the coefficient exceeds two standard errors*.

*R^2^ = Blocked-Error-R^2^ (Hayduk, 2006)*.

**Figure 3:**
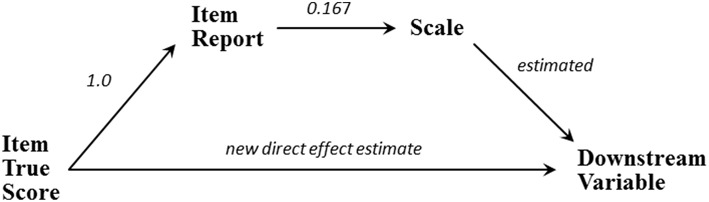
The direct and indirect effects of an item.

Each scale-bypassing effect corresponds to an indirect effect transmitted from the item's true score, through the item's observed score, to the scale, and finally to the same downstream variable, as depicted in Figure 3. Forming a scale by averaging items forces each item to have the same relatively small indirect effect on any specific downstream variable. For example, for Leadership the indirect effect of the Feedback item on Supportive Group is the product of the 1.0 effect connecting the item's true-score to the observed item, the 0.167 contribution of the item to the Leadership scale, and the scale's estimated 0.473 effect on Supportive Group; which is (1.0)(0.167)(0.473) = 0.079. This indirect effect is identical for all the scale's items because each item's indirect effect begins with 1.0, has the same middle value dictated by the number of averaged items, and employs the same estimated scale-effect on the downstream Supportive Group variable. Thus, each of the six Leadership items has an indirect effect on any specific downstream variable that is one-sixth the Leadership scale's effect on that downstream variable.

An effect leading directly from an item's true score to a downstream variable may either supplement or counteract this indirect effect. An item's total effect is the sum of its direct and indirect effects, so a positive direct effect supplements a positive indirect effect and indicates the item has a stronger impact on the downstream variable than can be accounted for by the scale alone. A negative direct effect counteracts a positive indirect effect and indicates the scale provides an unwarrantedly strong connection between the item and downstream variable. For Leadership three of the four direct effects of items on downstream variables are negative, indicating that requiring these items to work through the Leadership scale produces artificially and inappropriately strong estimates of these items' effects on the applicable downstream variables (Table 4). The lone positive direct effect indicates one item (Mentors) should be granted a stronger impact on a downstream variable (Like Working Here) than the Leadership scale permits.

**Table 4:** Effects bypassing the leadership scale in the amended Alberta model.

**Effect**	**Indirect effect of the item via Leadership in the *baseline* model**	**Indirect effect of the item via Leadership in the *amended* model**	**Direct effect of the item in the *amended* model**	**Direct plus indirect effect of the item in the *amended* model**
From Feedback to Supportive	0.069	0.079	−0.102	−0.023
From Success to Taken Seriously	0.072	0.076	−0.084	−0.008
From Calmly to Time for Extra	0.036	0.069	−0.206	−0.137
From Mentors to Like Work Here	0.034	0.020	0.082	0.102

*The causal variables are the item true scores*.

*The reported baseline indirect effect = (1.0) (0.167) (estimated scale effect in the Baseline model)*.

*The reported amended indirect effect = (1.0) (0.167) (estimated scale effect in the Amended model)*.

*The direct effects, the indirect effects, and the direct plus indirect effects are “basic effects” (Hayduk, 1987, p. 249) and do not include the enhancements introduced by effects cycling through the loops*.

The guaranteed-weak indirect effects of items acting through scales are susceptible to being overshadowed by effects leading directly from the items to downstream variables. All three negative direct item effects in the amended Leadership model, for example, are stronger than the items' small-positive effects carried through the Leadership scale. Two of these direct item effects essentially nullify the corresponding indirect effects, but the third produces a noticeable net negative (reversed) impact (Table 4). The Leadership scale's validity is clearly questioned whenever an item's direct effect nullifies or reverses an effect purportedly attributable to the scale containing that item. Direct effects substantially enhancing an item's indirect effect through the scale similarly question the scale (e.g., the direct effect from Mentoring to Like Working Here) because this also signals the scale's inability to appropriately represent the item's causal capabilities. Only four of 42 possible direct effects of the six items on the seven downstream variables are required in the enhanced Leadership model but these effects clearly recommend theoretical reconsideration of the Leadership scale. The involvement of several different scale items and several different outcome variables make the theory challenges somewhat awkward.

The two remaining coefficients added in creating the amended Leadership model lead to the “Leadership scale”—one from an exogenous variable (Have Enough Staff), the other from a downstream variable (Time To Do Something Extra). It is tempting but incorrect to think of these effects as explaining Leadership as originally conceptualized, for example by claiming that health care aides attribute sufficient/insufficient staff to superior/inferior unit leadership as originally scaled. This interpretation is inconsistent with the amended model's estimates because additional causes leading to the scale variable do not explain the original Leadership scale. The new effects redefine the scale such that it only partially corresponds to the original Leadership scale. The original scale was defined as

$$\begin{array}{l} \text{Original Leadership Scale}=\left(\text{average of six relevant items}\right). \end{array}$$

Retaining the same fixed item effects that defined the Leadership scale while adding a new variable's effect changes the equation to

$$\begin{array}{l} \text{New Leadership Scale​ = ​}(\text{average of six relevant items})\\ \text{ + }(\text{estimated effect of})\;(\text{a newly added cause})\\ \text{New Leadership Scale​ =​ }(\text{Original Leadership Scale})\\ \text{ + }(\text{estimated effect of})(\text{a newly added cause}). \end{array}$$

A predictor variable in an equation does not explain another predictor in that equation, so any additional cause does not explain the original scale, it redefines the scale. The original version of Leadership is transformed into new-Leadership where Enough Staff and Time for Something Extra become components of new-Leadership as opposed to “explaining” anything about Leadership as originally specified and defined. Explaining original Leadership would require explaining the items averaged to create the original Leadership scale.

The downstream variables will usually be included in the model because they are directly caused by the scale, so enhancing a model by adding an effect leading from a downstream variable back to the scale is likely to introduce a causal loop. The additional effect leading from Time for Something Extra to New-Leadership entangles New-Leadership in just such a loop (see Figure 2). Though somewhat unusual, causal loops are understandable and not particularly statistically problematic (Hayduk, 1987 Chapter 8; Hayduk, 1996 Chapter 3). A more fundamental concern is that even this single causal loop ensnares Leadership in a causal web that renders it impossible to define or measure Leadership without modeling the appropriate looped causal structure. A variable that was formerly an effect of Leadership becomes both a cause and effect of New-Leadership—and that new causal embeddedness renders standard measurement procedures inappropriate. Items that act as causes can be averaged to create scale scores but we currently have no way of creating scores for “scale” variables trapped in causal loops containing both their causes and effects. The only appropriate option is to place a “scale” like New-Leadership in a model respecting the relevant causal complexities. That stymies traditional scale score calculations even though it employs the same observed variables and permits valid investigation of the causal connections between the scale items, the scale, and the downstream variables.

We now briefly consider the fusion validity of the Leadership scale using data from health care aides in the Canadian province of Manitoba. The Manitoba model employs the same percentage of measurement error variance as in Alberta and is structured identically to the baseline Alberta model with the exception that the smaller of one pair of downstream reciprocal effects was provided a small fixed value (Supportive to Control, −0.150) to avoid underidentification—which results in the baseline Manitoba model having one more degree of freedom than the Alberta model. The Manitoba baseline Leadership model, like the Alberta baseline model, was highly significantly inconsistent with the data (Table 2). Amending the model by freeing one item's effect on a downstream variable (Calmly Handles to Observations Taken Seriously) and permitting the exogenous variable Enough Staff to influence “Leadership” resulted in a model that fit nearly as well as the amended Alberta model and with similar estimates (Tables 2, 3).

The small number of demanded alterations is comforting but the repeated requirement for an effect of the control variable Enough Staff on “Leadership” is particularly noteworthy. Two separate data sets report that “Leadership” as perceived by health care aides should be redefined to include Enough Staff in order to make the Leadership scale consistent with the evidence. The remaining alterations differ between the Alberta and Manitoba models, including the challenging loop-creating effect, and these clearly warrant additional investigation. But rather than pursuing the substantive details of these Leadership models, we turn to more general technicalities involved in assessing fusion validity.

### Technicalities, Extensions, and Potential Complexities

We developed fusion validity to investigate scales developed by researchers participating in TREC (Translating Research into Elder Care) studies of residents and care aides in long-term care facilities (Estabrooks et al., 2009a) and not as an intentional continuation or extension of specific statistical traditions. We thank one of our reviewers for encouraging us to report and reference connections between fusion validity and various threads within the statistical and methodological literature. Fusion validity's grounding in causal networks places it closer to the causal-formative (rather than composite-formative) indicators discussed by Bollen and Bauldry (2011), and fusion validity's dependence on context-dependent theory distances it from some components of traditional classical test theory. The inclusion of both a scale and its items within the same model provides an opportunity to reassess the points of friction evident in exchanges between Hardin (2017) and Bollen and Diamantopoulos (2017). The points are too diverse and complex for us to resolve, though we hope our comments below provide helpful direction.

Fusion validity's dependence on embedding the scale in an appropriate causal context raises potential technical as well as theoretical concerns. The baseline model may fit, or fail to fit, and either result may prove problematic. A fitting baseline model containing unreasonable estimates questions whether the control and downstream variables are sufficiently well-understood to be entrusted with scale adjudication. Nothing forbids a few mild modifications to initially-failing baseline models but it may be technically tricky to avoid inserting coefficients more appropriately regarded as scale-confronting. Reasonable modifications might rectify downstream variables' causal interconnections, or exogenous control variables' connections to the downstream variables, but ferreting out whether or not a modification questions the scale may prove difficult. For example, if a control variable correlates substantially with an item's true-scores the modification indices may equivocate between whether the control variable or the item effects a downstream variable, and thereby equivocate between whether the researcher is confronting scale-compatible or scale-incompatible evidence. Baseline models having complicated interconnections among the downstream variables, or unresolved issues with multiple indicators of control or downstream variables are likely to prove particularly challenging. Neophytes may have difficulty recognizing, let alone resisting, coefficients that could lead to inappropriately obtained model fit, especially knowing that persistent baseline model failure questions their scale. Validity requires consistency with our understandings, but when our modeled understandings (whether in a baseline or amended model) are problematic, concern for validity transmutes into concern for the fundamental commitments underlying scientific research.

Standardized residual covariances typically provide diagnostic direction, but they provided minimal assistance in fusion validity assessments because the scale latent variable and the item true-score latents have no direct indicators and consequently contribute only indirectly to the covariance residuals. Furthermore, the residual covariance ill fit among the scale items should be essentially zero because the model's structure nearly guarantees that the estimated covariances among the item true scores should reproduce the observed item covariances irrespective of the number or nature of the items' sources. This “guaranteed” perfect fit among the items might be thought of as a diagnostic limitation, but it is more appropriately thought of as convincingly demonstrating that fusion validity does not depend on the items having a common factor cause. The free covariances among the item true scores permit the items to reflect a single factor, but also permit the item true scores to reflect multiple different “factors.” Thus, fusion validity can assess scales created from both reflective and formative indicators (Bollen and Lennox, 1991). The issue addressed by fusion validity is not the source of the items but whether the items causally combine into a scale that is unidimensional in its production of downstream variables. Fusion validity is not about the dimensionality of the scale variable. The scale variable is unavoidably unidimensional no matter the number of constituent items or the number of “factors” producing those items. The issue is the *causal fidelity of fusing the potentially-diverse items* into a unidimensional variable capable of transmitting the potentially-diverse items' effects to the downstream variables.

If the baseline model fails after exhausting reasonable modifications, the focus switches to scale-questioning connections between specific items and the downstream variables, and/or additional effects leading to the scale in an amended model. Here the most useful diagnostics are the modification indices and expected parameter change statistics. A large, not merely marginally-significant, modification index for an item's effect on a downstream variable, combined with an implicationally-understandable expected parameter change statistic, would suggest including a coefficient speaking against the scale. The magnitude and sign of the expected parameter change statistic for an item's direct effect should be understandable in the context of the indirect effect that the item transmits through the scale as discussed in regard to Figure 3. A scale-bypassing effect speaks against the thoroughness of the encapsulation provided by the scale but if the world contains multiple indirect effect mechanisms (Albert et al., 2018), it might require both a direct item effect and the indirect effect acting through a fused scale. Unreasonably-signed scale bypassing effects speak more clearly against the scale.

If one specific item requires stronger (or weaker) effects on multiple downstream variables, and if the required effect adjustments are nearly proportional to the scale's effects, that might be accommodated by strengthening (or weakening) the item's fixed effect on the scale. For example, a substantial modification index corresponding to one item's fixed 0.167 effect leading to the Leadership scale might recommend constructing a weighted Leadership scale rather than the current average scale. Similarly, if the baseline model contained fixed unequal item weightings, large modification indices for some weights might recommend reweighting the items.

It should be clear that an amended model requiring a direct effect of an item's true-score on a downstream variable is not equivalent to, and should not be described as, having altered the item's contributions via the scale. Effects transmitted via the scale must spread proportionately to all the variables downstream from the scale. An effect leading from one item to a specific downstream variable disrupts the scale's proportional distribution requirement for that specific pairing of an item and downstream variable. The new direct effect also loosens (“partially frees”) the constraints on that item's effects via the scale on the other downstream variables because these other effects need no longer be rigidly proportional to this item's effect via the scale on the bypass-receiving downstream variable. The proportionality constraints on the other items' effects (via the scale) on the downstream variables are also slightly loosened by the scale-bypassing effect but the greater the number of items and scale-affected downstream variables the feebler the loosening of these constraints. Each additional scale-bypassing effect progressively, even if minimally, loosens the proportionality constraints on all the items' effects on the downstream variables via the scale. This suggests an accumulation of minor constraint relaxations resulting from multiple scale-bypassing effects in an amended model might constitute holistic scale-misrepresentation.

A substantial modification index might also be connected to the fixed zero variance assigned to the residual variable that causes the scale—namely the zero resulting from the absence of an error variable in the item-averaging equation constructing the scale. A substantial modification index here suggests some currently unidentified variable may be fusing with the modeled scale items, or that there are some other unmodeled common causes of the downstream variables. A scale known to be incomplete due to unavailability of some specific cause might warrant assigning the scale's residual variance a fixed nonzero value, or possibly a constrained value. The scale's residual variance might even be freed if sufficient downstream variables were available to permit estimation. A nonzero residual variance should prompt careful consideration of the missed-variable's identity. The potential freeing of the scale's residual variance clearly differentiates fusion validity from confirmatory composite analysis, which by definition forbids each composite from receiving effects from anything other than a specified set of indicators (Schuberth et al., 2018, p. 3). Indeed, the potential freeing of the scale's residual variance pinpoints a causal conundrum in confirmatory composite analysis—namely how to account for the covariance-parameters connecting composites without introducing any additional effects leading to any composite (Schuberth et al., 2018, Figure 5). This is rendered a non-issue by fusion validity's causal epistemological foundation. The relevant modeling alternatives will be context-specific but likely of substantial theoretical and academic interest.

The fixed measurement error variances on the observed items might also require modification but the implications of erroneous values of this kind are likely to be difficult to detect, and could probably be more effectively investigated by checking the model's sensitivity to alternative fixed measurement error variance specifications. Modeling the items' and/or scale's residual variables as independent latent variables (Hayduk, 1987, p.191-198) would provide modification indices permitting assessment of potential measurement error covariances paralleling the proposals of Raykov et al. (2017). Attending to modification indices, or moving to a Bayesian mode of assessment, would implicitly sidle toward exploration, which nibbles at the edges of validity, so especially-cautious and muted interpretations would likely be advisable.

Other technicalities might arise because the scale variable and the item true score variables have no direct indicators, which forces the related model estimates to depend on indirect causal connections to the observed indicators. The scale's effects on the downstream variables, for example, are driven by the observed covariances between the items' indicators and the indicators of the downstream variables because the scale's effects provide the primary (even if indirect) causal connections between these sets of observed indicators. And the covariances among the “indicatorless” item true scores will mirror the covariances of the observed item indicators because the true scores' covariances constitute the primary causal sources of these covariances. The absence of direct latent to indicator connections may produce program-specific difficulties, as when the indicatorless item true score latents stymied LISREL's attempts to provide start values for these covariances (Joreskog and Sorbom, 2016). This particular technicality is easily circumvented by providing initial estimates approximating the corresponding items' observed variances and covariances.

Related complexities may arise because programs like LISREL require modeling the observed items as perfectly measured latents (with λ = 1.0, and Θε = 0.0) as in Figure 1, which moves the measurement error variances into LISREL's Ψ matrix and places zero variances in Θε, thereby producing an expected and ignorable warning that Θε is not positive definite. This statistical annoyance arises because the measurement error variance in each item unavoidably contributes to the scale. This could be transformed into an interesting theoretical issue by considering that in some contexts it might be reasonable to think of this as “specific variance” which could be split into an item's measurement error variance dead-ending in the indicator (namely a non-zero Θε in LISREL) and another part indirectly contributing to the scale and downstream variables (as in the illustrated fixed Ψ specification). In the extreme, a fusion validity model might specify all the item measurement error variance as dead-ending in the indicators so the scale is created from fixed effects arriving from the items' true-scores. This would correspond to moving the fixed effects currently leading to the scale from the observed-items to the true-score items in Figure 1, and would permit investigating how a scale would function if it was purified of indicator measurement errors. This version of the fusion validity model would attain the epitome of scale construction—a scale freed from measurement errors—which is unattainable in contexts employing actual error-containing items. Contrasting the behavior of the “measurement error free” and “real” scales would permit assessing whether the unavoidable incorporation of items' measurement errors in the “real” scale introduces consequential scale degradation or interference.

It would be possible to simultaneously assess the fusion validity of two or more different scales constructed from a single set of items if the model contains downstream variables differentially responding to those scales. This opens an avenue for assessing Bollen and Bauldry (2011) differentiation between “covariates” and measures, and it provides a route to resolving the confusions plaguing formative indicators, partial least squares, and item parcels (Little et al., 2013; Marsh et al., 2013; Henseler et al., 2014; McIntosh et al., 2014). Importantly, factor score indeterminacy does not hinder fusion validity assessments. Indeed, if the items were modeled as being caused by a common factor (rather than as having separate latent causes as illustrated), fusion-validity modeling of the scale would provide a potentially informative estimate of the correlation between the factor and the scale (now factor scores).

We should also note that fusion validity surpasses composite invariance testing (Henseler et al., 2016): because fusion validity assessment is possible with a single group, because it employs as sophisticated a theory as the researcher can muster, and because validity supersedes mere reliability/invariance. Introducing a longitudinal component to a fusion validity model would even permit differentiating “specificity” from “error” (Raykov and Marcoulides, 2016a) if the fusion validity model incorporates factor structuring of the items. In general, replacing items with parcels disrupts the item-level diagnostics potentially refining fusion validity models, and hence is not advised. A reviewer noted that attention to non-linearities might “introduce more flexibility (and fun)” into fusion validity. We agree—but quite likely “fun” for only the mathematically-inclined (Song et al., 2013).

Fusion validity's theory-emphasis does not end with formulation of appropriate baseline and amended models—it may extend into the future via consideration of what should be done next. For example, one author (*CE*) was concerned that the demand for parsimony during data collection resulted in omission of causes of leadership, and she was uneasy about employing downstream latents having single indicators instead of similarly named scales having multiple indicators. These seemingly methodological concerns transform into theory-options as one considers exactly how a supposedly-missed cause should be incorporated in an alternative baseline model—namely is the missed variable a control variable, a downstream variable, or possibly an instantiation of the scale's residual variable? These have very different theoretical and methodological implications. Similar detailed theoretical concerns arise from considering how an additional-scale, or multiple indicators used by others as a scale, should be modeled by a researcher investigating a focal scale such as Leadership. Fusion validity models are unlikely to provide definitive-finales for their focal scales but rather are likely to stand as comparative structural benchmarks highlighting precise and constructible theoretical alternatives. An advance in theory-precision is likely, irrespective of the focal scale's fate.

## Discussion and Conclusions

A scale's fusion validity is assessed by simultaneously modeling the scale and its constituent items in the context of appropriate theory-based variables. Fusion validity presumes the items were previously assessed for sufficient variance, appropriate wordings, etcetera, and that a specific scale-producing procedure exists or has been proposed (whether summing, averaging, factor score weightings, or conjecture). This makes the scale's proximal causal foundations known because the researcher knows how they produce, or anticipate producing, scale values from the items, but whether the resultant scale corresponds to a unidimensional world variable appropriately fusing and subsequently dispensing the items' effects to downstream variables awaits fusion validity assessment.

Fusion validity circumvents the data collinearity between a scale and its constituent items by employing only the items as data while incorporating the scale as a latent variable known through its causal foundations and consequences. The scale is modeled as encapsulating and fusing the items, and as subsequently indirectly transmitting the items' impacts to the downstream variables. An item effect bypassing the scale by running directly to a downstream variable signals the scale's inability to appropriately encapsulate that item's causal powers.

The fixed effects leading from the items to the scale are dictated by the item averaging, summing, or weighting employed in calculating the scale's values. The effects leading from the scale to the downstream variables are unashamedly, even proudly, theory-based because validity depends upon consistency with current theoretical understandings (Cronbach and Meehl, 1955; Hubley and Zumbo, 1996; American Educational Research Association, 2014). After reviewing scale assessments in multiple areas, Zumbo and Chan observed that “by and large, validation studies are not guided by any theoretical orientation, validity perspectives or, if you will, validity theory” (Zumbo and Chan, 2014, p. 323). The unavoidable collinearity between item and scale data ostensibly hindered checking the synchronization between items, scales, and theory-recommended variables—a hindrance overcome by the fusion validity model specification presented here.

It is clear how items caused by a single underlying factor might fuse into a unidimensional scale. The consistent true-score components of the items accumulate and concentrate the underlying causal factor's value while random measurement errors in the items tend to cancel one another out. The simplicity and persuasiveness of this argument switched the historical focus of scale validity assessments toward the factor structuring of the causal source of the items and away from the assessment of whether some items fuse to form a scale entity. Fusion validity examines whether the items fuse to form a unitary variable irrespective of whether or not the items originate from a common causal factor. That is, fusion validity acknowledges that the world's causal forces may funnel and combine the effects of items even if those items do not share a common cause. It is possible for non-redundant items failing to satisfy a factor model to nonetheless combine into a unidimensional scale displaying fusion validity. For example, the magnitude of gravitational, mechanical, and frictional forces do not have a common factor cause, yet these forces combine in producing the movement of objects. The causal world might similarly combine diverse psychological or social attributes into unidimensional entities such as Leadership ability, or the like. Given that diversity among the items' causes does not dictate whether or not those items fuse, it remains possible for items failing to comply with a factor model to nonetheless fuse into valid scales—though the fusing is “not guaranteed” and requires validation.

And the reverse is also possible. Items having a common cause and satisfying the factor model may, or may not, fuse into valid scales. That is, items sharing a common cause do not necessarily have common effects. For example, the number of sunspots is a “latent factor” that causes both the intensity of the northern lights and the extent of disruption to electronic communications but we know of no causally downstream variable responding to a fused combination of northern light intensity and communication disruption. In brief, fusion validity focuses on whether the items' effects combine, meld, or fuse into an effective unidimensional scale entity irrespective of the nature of the items' causal foundations. If a researcher believes their items share a common factor cause and also fuse into a scale dimension, it is easy to replace the item true-score segment of the fusion validity model with a causal factor structure. Such a factor-plus-fusion model introduces additional model constraints and is more restrictive than the illustrated fusion validity model specification. The appropriateness of the additional factor-structure constraints could be tested via nested-model χ^2^-difference testing, and might be informative, but would not be required for fusion validity. Fusion validity can therefore be applied to both reflective and formative indicators.

Evidence confronting a scale arises when a failing baseline model must be amended: by introducing item effects bypassing the scale on the way to downstream variables, by introducing additional effects leading to the scale, by altering the fixed effects constituting the scale's calculation, or by altering the error variance specifications. An effect leading directly from an item to a downstream variable alters the understanding of the scale irrespective of whether that effect supplements or counteracts the item's indirect effect through the scale. Either way, the scale is demonstrated as being incapable of appropriately encapsulating the item's causal consequences, and hence retaining both the item and scale may be required for a proper causal understanding. An item effect bypassing the scale does not necessarily devastate the scale because it is possible for several items to fuse into an appropriate scale entity having real effects and yet require supplementation by individual item effects. Items having direct effects on downstream variables that cancel out or radically alter the item's indirect effect via the scale are more scale-confronting. Scale-bypassing effects and other model modifications encourage additional theory precision—precision which is likely to constitute both the most challenging and the most potentially-beneficial aspect of fusion validity assessment.

Amending the baseline model by introducing an additional effect leading to the scale variable—namely an effect beyond the originally scale-defining item effects—produces a new and somewhat different, but potentially correct, scale variable. The new effect does not explain the original scale. Both the original scale and new-scale are fully explained because both scales typically have zero residual error variance. They are just different fully explained variables which possess and transmit somewhat different effects. The new scale variable may retain the ability to absorb and transmit the original items' effects to the downstream variables but the new scale is also capable of absorbing and transmitting the actions of the additional causal variable. The researcher's theory should reflect a scale's changing identity. Both theory and methods are likely to be challenged by attempting to expunge the old scale scores from the literature—especially since the new scale's scores would not be calculable in existing data sets lacking the new scale-defining variable.

Both theory and methods are likely to be more strongly challenged if model alteration requires effects leading to the scale from downstream variables because such effects are likely to introduce causal loops. Loops provide substantial, though surmountable, theory challenges (Hayduk, 1987, 1996, 2006; Hayduk et al., 2007) but they introduce especially difficult methodological complications because there is no standard procedure for obtaining values for scales entangled in loops containing their effects. A model can contain as many equations as are required to properly model looped causal actions but the single equation required for calculating a scale's scores becomes unavoidably misspecified if the equation contains one of the scale's effects as a contributory component. If a substantial modification index calls for a loop-producing effect that effect would likely be identified. In contrast, theory-proposed looped effects may prove more difficult to identify (Nagase and Kano, 2017; Wang et al., 2018; Forre and Mooij, 2019).

The requirement that valid scales function causally appropriately when embedded in relevant theoretical contexts implicitly challenges factor models for having insufficient latent-level structure to endorse scale validity. Indeed, fusion validity assessment supersedes numerous factor analytic “traditions.” The lax model testing evident in even recent factor analysis texts contrasts with the careful testing required for the baseline and enhanced fusion validity models (Hayduk, 2014a,b; Brown, 2015). And if a baseline or enhanced model is inconsistent with the downstream variables, researchers steeped in traditional factor practices are likely to reflexively attempt to “fix” the model by inserting indicator error covariances or by deleting indicators, rather than retaining the indicators and adding theory-extending latents. Adding latents implicitly challenges the multiple indictors touted by factor analysis because adding latents while retaining the same indicators sidles toward single indicators (Hayduk and Littvay, 2012). Researchers from factor analytic backgrounds are likely to find it comparatively easy to sharpen their model testing skills but will probably encounter greater difficulty pursuing theoretical alternatives involving effects among additional similar latent variables, or appreciating how items having diverse causal backgrounds might nonetheless combine into an effective unidimensional causal entity—such as leadership, trust, stress, or happiness. The tight coordination between theory and scale validity assessment provides another illustration of why measurement should accompany, not precede, theoretical considerations (Cronbach and Meehl, 1955; Hayduk and Glaser, 2000a, Hayduk and Glaser, 2000b).

Scales were traditionally justified as more reliable than single indicators, and as easier to manage than a slew of indicators. Both these justifications crumble however, if the scale's structure is importantly causally misspecified, because invalidity undermines reliability, and because a causal-muddle of indicators cannot be managed rationally. In medical contexts, for example, it is unacceptable to report a medical trial's outcome based on a problematic criterion scale, but equally unacceptable to throw away the data and pretend the scale-based trial never happened. This dilemma underpins the call for CONSORT (the Consolidated Standards for Reporting Trials) to instruct researchers on how to proceed if a scale registered as a medical trial's criterion measure is found to misbehave (Downey et al., 2016). The impact of some assumption violations on scale reliability have been addressed for factor-structured models (Raykov and Marcoulides, 2016b) but if the causal world is not factor structured, the nature and utility of “reliability” remains obscure. And what constitutes “criterion validity” (Raykov et al., 2016) if both the criterion and the scale happen to be involved in a causal loop? Ultimately, avoiding iatrogenic consequences requires a proper causal, not merely correlational, understanding of the connections linking the items, the scale, the downstream variables, and even the control variables. Pearl and Mackenzie (2018) and Pearl (2000) present clear and systematic introductions to thinking about causal structures and why control variables deserve consideration. One of our reviewers pointed us toward a special issue of the journal *Measurement* focused on causal indicators and issues potentially relating to fusion validity. We disagree with enough points in both the target article by Aguirre-Urreta et al.'s (2016) and the appended commentaries that we recommend these exchanges as a practice-exam for anyone considering investigating a fusion validity model. Try to follow the consequences of the Aguirre-Urreta et al. (2016) simulation having: (a) employed causal indicators that do not require any control variables, and (b) having used causal indicators that are forbidden effects bypassing the scale variable. It should also prove instructive to notice the emergent focus on measurement's connection to substantive theory—and not just measurement traditions.

The assessment of fusion validity illustrated above slightly favors the scale by initially modeling the scale's presumed effects, and by permitting baseline model modifications which potentially, even if inadvertently, assist the scale. A scale-unfriendly approach might begin with a baseline model permitting some scale-bypassing item effects, while excluding all the scale's effects on the downstream variables until specific scale effects are demanded by the data. However done, models assessing whether a set of items fuse to form a scale will depend on theory, will focus attention on theory, and will provide opportunities to correct problematic theoretical commitments.

Fusion validity shares traditional concerns for item face validity and methodology but requires variables beyond the items included in the scale—specifically variables causally downstream from the postulated scale but possibly control variables which may be upstream of the items. Fusion validity permits but does not require that the scaled items have a common factor cause, or even that the items correlate with one another.

Traditional formulations make reliability a prerequisite for validity but some forms of reliability are not a prerequisite for fusion validity because fusion validity does not share a factor-model basis. It does require that the items fuse or meld in forming the scale according to the researcher's specifications. Consequently, just as construct validity cannot “be expressed in the form of a single simple coefficient” (Cronbach and Meehl, 1955, p. 300), fusion validity assessment does not produce one single coefficient's value and instead depends on the researcher's facility with structural equation modeling to assess the scale's coordination with whatever substantive variables are required by theory. This means the researcher must be as attentive to the possibility of faulty theory as to faulty scaling—which seems to be an unavoidable concomitant of the strong appeal to theory required by seeking validity. Fusion validity's inclusion in the model of theory-based variables along with both the items and scale permits many assessments unavailable to traditional analyses, and potentially recommends correspondingly diverse theory, scale, and item improvements. Complexity abounds, so only those strong in both their theory and structural equation modeling need apply.

Embedding a scale in deficient theory will highlight the deficiencies, while embedding a scale in trustworthy theory will provide unparalleled validity assessments. Fusion validity assessment does not guarantee progress but provides a way to investigate whether our scales coordinate with our causal understandings, and a way to check whether traditional scale assessments have served us well.

## Availability of data and materials

The data analyzed in this study are from care aides in Alberta and Manitoba collected in 2014-2015 and are archived by the Translating Research into Elder Care (TREC) team at the University of Alberta. TREC is a pan-Canadian applied longitudinal (2007-ongoing) health services research program in residential long term care. The TREC umbrella covers multiple ethics-reviewed studies designed to investigate and improve long term care. The appended LISREL syntax contains the covariance data matrix sufficient for replicating the Alberta estimates or estimating alternative models.

## Ethics Statement

Ethics approval was obtained by the Translating Research in Elder Care team from both universities and all the institutions and participants participating in the reported studies.

## Author Contributions

LH conceived the analytical procedure, conducted the analyses, wrote the draft article, and revised the article incorporating coauthor suggestions. CE and MH critically assessed the article and suggested revisions. All authors contributed to manuscript revision, read and approved the submitted version.

### Conflict of Interest Statement

The authors declare that the research was conducted in the absence of any commercial or financial relationships that could be construed as a potential conflict of interest.

## Abbreviations

LISREL, Mplus: and AMOS are structural equation modeling programs
TREC: Translating Research into Elder Care
ACT: Alberta Context Tool
CONSORT: Consolidated Standards for Reporting Trials.
