# Supplementary material for: Fusion Validity: Theory-Based Scale Assessment via Causal Structural Equation Modeling
Source: Front Psychol. 2019 Jun 4;10:1139. doi: 10.3389/fpsyg.2019.01139 (PMC6559122; doi:10.3389/fpsyg.2019.01139)
Supplement: Supplementary file 1 [file Data_Sheet_1.docx]

# **Appendix:** **LISREL** **Model Syntax for TREC Leadership for Alberta Care Aides**

Title ...\Fusion Validity Leadership

! \...\LeadershipAlbertaBaseline+AmendedAppendix spl

! This syntax produces the Baseline Leadership model for Alberta, using LISREL 9.1

! (Joreskog and Sorbom, 2016).

! Deleting two ! marks documented below, introduces the six new estimates

! that create the Amended Leadership model.

DA NI=23 NO=1610 MA=CM

! NO is minimum pairwise N, max possible was 1620

CM FU

.068 .053 -.009 .003 -.002 .011 .007 .000 -.001 -.001 -.003 -.004 .006 .003

-.003 -.006 .001 -.014 -.016 .009 .002 -.006 -.005

.053 4.795 -.072 -.005 -.011 -.025 -.023 -.005 -.119 -.014 -.013 -.089

-.044 -.075 -.082 -.013 -.024 -.122 -.023 -.133 .102 -.088 .139

-.009 -.072 .228 .009 .003 .000 .025 .037 .038 .073 .074 .066 .079 .026 .036 .068 -.010 .118 .122 -.077 .056 .014 .007

.003 -.005 .009 .676 .210 .244 .258 .260 .254 .144 .113 .121 .135 .106 .097 .152 .111 .102 .102 -.124 .118 .005 .039

-.002 -.011 .003 .210 .596 .233 .210 .218 .215 .128 .117 .110 .137 .094 .113 .106 .074 .114 .093 -.129 .135 .016 .023

.011 -.025 .000 .244 .233 .592 .307 .274 .290 .180 .130 .140 .163 .123 .112 .157 .110 .152 .070 -.152 .145 .011 .050

.007 -.023 .025 .258 .210 .307 .536 .309 .302 .193 .165 .159 .178 .148 .138 .170 .098 .173 .146 -.194 .151 .018 .093

.000 -.005 .037 .260 .218 .274 .309 .583 .354 .179 .179 .179 .177 .123 .118 .185 .098 .183 .167 -.178 .187 .017 .049

-.001 -.119 .038 .254 .215 .290 .302 .354 .683 .180 .175 .178 .204 .136 .136 .200 .095 .213 .200 -.231 .182 .019 .026

-.001 -.014 .073 .144 .128 .180 .193 .179 .180 .773 .321 .264 .288 .168 .224 .224 .153 .275 .219 -.295 .237 .020 .087

-.003 -.013 .074 .113 .117 .130 .165 .179 .175 .321 .624 .249 .272 .191 .160 .205 .125 .231 .179 -.258 .199 .010 .017

-.004 -.089 .066 .121 .110 .140 .159 .179 .178 .264 .249 .593 .336 .175 .199 .217 .099 .291 .237 -.273 .221 .025 .108

.006 -.044 .079 .135 .137 .163 .178 .177 .204 .288 .272 .336 .621 .230 .208 .236 .110 .292 .256 -.300 .257 .007 .096

.003 -.075 .026 .106 .094 .123 .148 .123 .136 .168 .191 .175 .230 .397 .188 .162 .099 .172 .133 -.148 .147 .014 .072

-.003 -.082 .036 .097 .113 .112 .138 .118 .136 .224 .160 .199 .208 .188 .672 .161 .133 .209 .187 -.167 .182 .032 .100

-.006 -.013 .068 .152 .106 .157 .170 .185 .200 .224 .205 .217 .236 .162 .161 .608 .215 .247 .186 -.263 .214 .014 .110

.001 -.024 -.010 .111 .074 .110 .098 .098 .095 .153 .125 .099 .110 .099 .133 .215 .601 .098 .064 -.146 .117 .015 .018

-.014 -.122 .118 .102 .114 .152 .173 .183 .213 .275 .231 .291 .292 .172 .209 .247 .098 1.567 .579 -.797 .298 .091 .260

-.016 -.023 .122 .102 .093 .070 .146 .167 .200 .219 .179 .237 .256 .133 .187 .186 .064 .579 1.409 -.546 .290 .091 .172

.009 -.133 -.077 -.124 -.129 -.152 -.194 -.178 -.231 -.295 -.258 -.273 -.300

-.148 -.167 -.263 -.146 -.797 -.546 4.270 -.496 -.026 -.650

.002 .102 .056 .118 .135 .145 .151 .187 .182 .237 .199 .221 .257 .147 .182 .214 .117 .298 .290 -.496 .556 .012 .113

-.006 -.088 .014 .005 .016 .011 .018 .017 .019 .020 .010 .025 .007 .014 .032 .014 .015 .091 .091 -.026 .012 .245 .021

-.005 .139 .007 .039 .023 .050 .093 .049 .026 .087 .017 .108 .096 .072 .100 .110 .018 .260 .172 -.650 .113 .021 2.725

LA

VAR022 VAR023 VAR024

VAR052 VAR053 VAR054 VAR055 VAR056 VAR057

VAR058 VAR059 VAR060 VAR061 VAR062 VAR063

VAR111 VAR113 VAR129 VAR136 VAR159 VAR223

VAR005R AggreSum

!Select the indicators in the proper sequence.

SE

VAR059 VAR063 VAR111 VAR113 VAR136 VAR223 VAR159

VAR022 VAR023 VAR024

VAR005R VAR129 AggreSum

VAR052 VAR053 VAR054 VAR055 VAR056 VAR057 /

MO NY=19 NX=0 NE=26 NK=0 LY=FU,FI BE=FU,FI PS=SY,FI TE=SY,FI

! E# in the labels below refer to Eta# in LISREL notation.

LE

E1SupGrp E2Contro E3ObsTak E4Talk E5Extra E6LikeHr E7BurnOu

E8FeedBk E9Succes E10Calm E11Liste E12Mento E13Resol

E14Leadr

E15Sex E16Age E17Engli E18Proft E19Staff E20Aggre

E21V052 E22V053 E23V054 E24V055 E25V056 E26V057

VA 1.0 LY(1,1) LY(2,2) LY(3,3) LY(4,4) LY(5,5) LY(6,6) LY(7,7)

VA 1.0 LY(8,15) LY(9,16) LY(10,17) LY(11,18) LY(12,19) LY(13,20)

VA 1.0 LY(14,21) LY(15,22) LY(16,23) LY(17,24) LY(18,25) LY(19,26)

!Creating the Leadership Scale variable

VA 0.16667 BE(14,21) BE(14,22) BE(14,23) BE(14,24) BE(14,25) BE(14,26)

!Differentiating the True values from Reported values

VA 1.0 BE(21,8) BE(22,9) BE(23,10) BE(24,11) BE(25,12) BE(26,13)

FR BE(1,14)

FR BE(2,14)

FR BE(3,14)

FR BE(4,14)

FR BE(5,14)

FR BE(6,1)

FR BE(6,2)

FR BE(6,3)

FR BE(6,4)

FR BE(6,5)

FR BE(6,14)

FR BE(7,1)

FR BE(7,2)

FR BE(7,3)

FR BE(7,4)

FR BE(7,5)

FR BE(7,6)

FR BE(7,14)

FR BE(1,2) BE(1,3) BE(1,4) BE(1,17)

FR BE(2,1) BE(2,4)

FR BE(3,4) BE(3,17)

FR BE(4,3) BE(4,17)

EQ BE(3,4) BE(4,3)

ST 0.01 BE(3,4)

FR BE(5,17) BE(5,18) BE(5,19)

FR BE(6,7) BE(6,16) BE(6,17) BE(6,18) BE(6,19) BE(6,20)

FR BE(7,6)

EQ BE(6,7) BE(7,6)

ST -0.01 BE(6,7)

FR BE(7,18) BE(7,19) BE(7,20)

! The Amended Leadership model can be created by deleting the

! two ! below that result in six effects being added

! to the Baseline Leadership model.

!FR BE(14,5) BE(14,19)

!FR BE(1,8) BE(3,9) BE(5,10) BE(6,12)

FR PS(1,1)

FR PS(2,2)

FR PS(3,3)

FR PS(4,4)

FR PS(5,5)

FR PS(6,6)

FR PS(7,7)

FR PS(8,8)

FR PS(9,8) PS(9,9)

FR PS(10,8) PS(10,9) PS(10,10)

FR PS(11,8) PS(11,9) PS(11,10) PS(11,11)

FR PS(12,8) PS(12,9) PS(12,10) PS(12,11) PS(12,12)

FR PS(13,8) PS(13,9) PS(13,10) PS(13,11) PS(13,12) PS(13,13)

ST 0.20 PS(9,8)

ST 0.20 PS(10,8) PS(10,9)

ST 0.20 PS(11,8) PS(11,9) PS(11,10)

ST 0.20 PS(12,8) PS(12,9) PS(12,10) PS(12,11)

ST 0.20 PS(13,8) PS(13,9) PS(13,10) PS(13,11) PS(13,12)

! PS(14,14) IS ZERO FOR LEADERSHIP

FR PS(15,8) PS(15,9) PS(15,10) PS(15,11) PS(15,12) PS(15,13)

FR PS(16,8) PS(16,9) PS(16,10) PS(16,11) PS(16,12) PS(16,13)

FR PS(17,8) PS(17,9) PS(17,10) PS(17,11) PS(17,12) PS(17,13)

FR PS(18,8) PS(18,9) PS(18,10) PS(18,11) PS(18,12) PS(18,13)

FR PS(19,8) PS(19,9) PS(19,10) PS(19,11) PS(19,12) PS(19,13)

FR PS(20,8) PS(20,9) PS(20,10) PS(20,11) PS(20,12) PS(20,13)

FR PS(15,15)

FR PS(16,15) PS(16,16)

FR PS(17,15) PS(17,16) PS(17,17)

FR PS(18,15) PS(18,16) PS(18,17) PS(18,18)

FR PS(19,15) PS(19,16) PS(19,17) PS(19,18) PS(19,19)

FR PS(20,15) PS(20,16) PS(20,17) PS(20,18) PS(20,19) PS(20,20)

! ATTITUDE indicators given 5% measurement error variance in PS

VA 0.0338 PS(21,21)

VA 0.0298 PS(22,22)

VA 0.0296 PS(23,23)

VA 0.0268 PS(24,24)

VA 0.02915 PS(25,25)

VA 0.03415 PS(26,26)

VA 0.0312 TE(1,1)

VA 0.0336 TE(2,2)

VA 0.0304 TE(3,3)

VA 0.03005 TE(4,4)

VA 0.07045 TE(5,5)

VA 0.0278 TE(6,6)

VA 0.2135 TE(7,7)

!EXOGENOUS indicators given specific % measurement error variances

VA 0.00068 TE(8,8) ! 1% error in sex.

VA 0.23975 TE(9,9) ! 5% error in age

VA 0.0114 TE(10,10) ! 5% error in First language English

VA 0.0 TE(11,11) ! 0.0% error in for profit or not

VA 0.07835 TE(12,12) ! 5% error in enough staff

VA 0.13625 TE(13,13) ! 5% error in Aggressive Acts Sum

OU ML ALL ND=3
